# RELATIONSHIP OF AGING KNOWLEDGE TO AGE BIAS VARIES ACROSS KNOWLEDGE DOMAIN FOR DOCTORAL PSYCHOLOGY TRAINEES

**DOI:** 10.1093/geroni/igae098.3258

**Published:** 2024-12-31

**Authors:** Benjamin Johnson, Grace Caskie

**Affiliations:** Lehigh University, Bethlehem, Pennsylvania, United States; Lehigh University, Bethlehem, Pennsylvania, United States

## Abstract

Doctoral psychology trainees often express low interest in clinical work with older adults, which may be attributed to ageist stereotypes they hold. Levy’s (2018) PEACE model states that one of the two most effective ways to reduce age bias is to increase accuracy of knowledge about aging. Few studies have examined psychology trainees’ aging knowledge or whether it relates to their age bias. We assessed the aging knowledge of 192 doctoral psychology trainees (aged 21-30 years), using Palmore’s Facts on Aging Quiz (FAQ), which includes three domains (biological, psychological, social). We also investigated the relationship of trainees’ aging knowledge to age bias (measured by FSA, ASD, and AmbAS). On average, trainees answered 13 FAQ items correctly (52%), indicating low aging knowledge. Trainees had more accurate aging knowledge in biological (M=70%) than psychological (M=60%) or social (M=40%) domains. They were significantly more likely (p<.001) to respond incorrectly to 10 facts and correctly to 11 facts; the social domain showed the most incorrect knowledge. Trainees’ total aging knowledge was not significantly correlated with any age bias measure; however, greater biological aging knowledge was positively correlated with more age bias (FSA and AmbAS), while greater psychological and social aging knowledge were negatively correlated with all age bias measures. Thus, to reduce age bias by increasing aging knowledge, interventions may need to be domain-specific. Additionally, psychology training may overemphasize biological aging and physical decline (i.e., medical model), suggesting interventions address other aspects of aging and focus on age-related gains in addition to losses.

